# Twenty-Five Years of Colorectal Cancer Screening in the Czech Republic: National Registry Trends in Program Performance and Cancer Burden

**DOI:** 10.3390/cancers18193082

**Published:** 2026-09-23

**Authors:** Stepan Suchanek, Monika Ambrozova, Tomas Grega, Renata Chloupkova, Ondrej Majek, Bohumil Seifert, Petr Kocna, Jan Bures, Ladislav Dusek, Miroslav Zavoral

**Affiliations:** 1 Department of Medicine, 1st Faculty of Medicine, Charles University, Military University Hospital Prague, U Vojenske Nemocnice 1200, 169 02 Prague, Czech Republic; tomas.grega@uvn.cz (T.G.); bures.jan@uvn.cz (J.B.); miroslav.zavoral@uvn.cz (M.Z.); 2 Department of Gastrointestinal Oncology, Military University Hospital Prague, U Vojenske Nemocnice 1200, 169 02 Prague, Czech Republic; 3 Institute of Biostatistics and Analyses, Faculty of Medicine, Masaryk University, 601 77 Brno, Czech Republic; monika.ambrozova@uzis.cz (M.A.); renata.chloupkova@uzis.cz (R.C.); ondrej.majek@uzis.cz (O.M.); ladislav.dusek@uzis.cz (L.D.); 4 National Screening Centre, Institute of Health Information and Statistics of the Czech Republic, 128 00 Prague, Czech Republic; 5 Institute of General Practice, 1st Faculty of Medicine, Charles University, Albertov 7, 128 01 Prague, Czech Republic; bohumil.seifert@lf1.cuni.cz; 6 Institute of Medical Biochemistry and Laboratory Diagnostics, 1st Faculty of Medicine, Charles University, General Faculty Hospital, U Nemocnice 2, 128 08 Prague, Czech Republic; petr.kocna@lf1.cuni.cz

**Keywords:** colorectal cancer, population-based screening, fecal immunochemical test, colonoscopy, early detection of cancer, quality indicators, Czech Republic, cancer registries

## Abstract

**Background/Objectives:** Colorectal cancer (CRC) incidence and mortality in the Czech Republic ranked among the highest worldwide for decades. The performance of the national screening program over 25 years is reported alongside concurrent population trends. **Methods:** In this retrospective population-based analysis, registry and health-insurance data on program indicators for 2006–2025 were analyzed by calendar year and screening pathway, together with national incidence and mortality for 1977–2024. Introduced in 2000 with guaiac fecal occult blood testing, the program added screening colonoscopy in 2009, completed the transition to fecal immunochemical testing (FIT) by 2014, and has used quantitative FIT only since 2020. The target age was 50 and over, redefined as 45–74 from 2026. **Results:** Among 653,825 preventive colonoscopies, at least one adenoma was detected in 251,974 examinations and cancer, including carcinoma in situ, in 16,575. The positive predictive value for adenoma after a positive fecal test rose from 29.6% to 48.3%, and the adenoma detection rate at screening colonoscopy from 25.3% to 32.6%. Cancer detection fell from 6.3% to 2.8% and from 1.8% to 0.5%, respectively. Coverage by screening modalities was 33.7%, and 41.0% including examinations outside the program. Six-month compliance with colonoscopy after a positive FIT was 58.0%. This figure counts colonoscopy under any procedure code and therefore measures whether colonoscopy was performed rather than whether it was reported under the dedicated pathway code. Of all patients with a positive FIT, 37.6% underwent colonoscopy reported under the dedicated FOBT+ colonoscopy code, a subset of the overall 58.0%. Waiting time for FOBT+ colonoscopy was 80 days on average. Age-standardized incidence and mortality were 31.7% and 50.0% lower in 2022–2024 than in 1998–2000. The declines were not uniform across age: the incidence decline was most pronounced at ages 75 and older, whereas the mortality decline was concentrated at ages 50 to 74. **Conclusions:** Sustained organized screening coincided with a substantial decline in one of the world’s highest CRC burdens, although its earlier part preceded program expansion. Coverage and completion of FOBT+ colonoscopy remain the principal implementation challenges.

## Simple Summary

Colorectal cancer has been one of the greatest cancer burdens in the Czech Republic for decades, with incidence and mortality among the highest recorded worldwide. Introduced in 2000, the national screening program was initially opportunistic and later transformed into population-based screening. Registry data described program performance in 2006–2025, and national incidence and mortality in 1977–2024. Among 653,825 preventive colonoscopies, at least one adenoma was detected in 251,974 examinations and cancer, including carcinoma in situ, in 16,575. Stage was recorded for only part of the cancers detected, so no conclusion can be drawn about a shift towards earlier stages. Since the program was launched in 2000, age-standardized incidence has fallen by about 32% and mortality by about 50%. Because individual screening histories were not linked to cancer outcomes, the contribution of screening to this decline cannot be determined. In 2025 one third of the target population was covered by screening, and 58% of people with a positive test had a follow-up colonoscopy within six months. Raising participation and shortening that wait are the program’s main challenges.

## 1. Introduction

Colorectal cancer (CRC) is the third most diagnosed cancer worldwide and the second leading cause of cancer death. In 2024 it accounted for 9.9% of new cancer cases and 9.4% of cancer deaths worldwide, with Europe among the world’s regions of highest incidence [1]. The Czech Republic carried one of the greatest burdens globally for decades. CRC mortality remains distinctly elevated in the European comparison, at 32 per 100,000 in 2021, 19% above the European Union (EU) average. Estimated incidence has converged: it is now close to the EU average in men, 94 against 93 per 100,000, and lies 9% below it in women [2].

The biological rationale for screening rests on the adenoma–carcinoma sequence. Most colorectal cancers develop from precancerous adenomatous polyps, whose endoscopic removal prevents progression to invasive cancer [3]. This protracted natural history creates a window for intervention. Endoscopic therapy can interrupt carcinogenesis, and fecal occult blood testing can detect occult hemorrhage from advanced adenomas or early cancers. Colonoscopic polypectomy has been associated with lower subsequent CRC incidence [4] and, on long-term follow-up, with lower CRC-related mortality [5]. Randomized trials of guaiac fecal occult blood testing (gFOBT) demonstrated reductions in CRC-specific mortality of 15% to 33% [6,7,8]. Organized programs differ from opportunistic testing: defined target populations are systematically invited, standardized protocols are applied, and quality is monitored across the pathway.

The Czech National CRC Screening Program was launched in 2000, following earlier national pilot projects. It was among the earlier nationwide CRC screening initiatives in Europe, and was subsequently transformed from opportunistic into population-based screening with the introduction of personal invitations in 2014 [9]. Its 25-year record coincides with the consolidation of organized screening as a cornerstone of European cancer policy [10,11]. The aim of this study was to describe the organization, technical evolution, quality, and coverage of the program, together with the population-level trends observed alongside it, and to situate its performance in the European context.

## 2. Materials and Methods

### 2.1. Data Sources

This is a retrospective, population-based analysis of routinely collected national registry and health-insurance data for 2006–2025. It is designed as a repeated cross-sectional description of annual program indicators, alongside a population-level time series of national cancer incidence and mortality, in which the unit of analysis is the population rather than the individual. Four data sources were used, three registries of the National Health Information System and national mortality statistics, and the source of each result is identified in the corresponding table or figure legend. Colonoscopy performance indicators derive from the Registry of Preventive Colonoscopies, a structured electronic reporting system aligned with the European performance measures for lower gastrointestinal endoscopy [12]. It was established in 2007, with records for 2006 entered retrospectively. Program-level colonoscopy indicators are therefore available from 2006, although the program has operated since 2000. The twenty-five years referred to throughout denote the duration of the program since its launch in 2000; the registry-based indicators cover 2006–2025 and the population-level series of incidence and mortality covers 1977–2024. The registry was administered until 2024 by the Institute of Biostatistics and Analyses, Faculty of Medicine, Masaryk University. From 2025 it has been maintained within the National Registry of Preventive and Screening Examinations. The data export used here is dated 26 February 2026, by which time reporting for 2025 was complete.

Program coverage, screening test volumes, follow-up compliance, and waiting times for colonoscopy after a positive fecal immunochemical test (FIT) derive from the National Registry of Reimbursed Health Services, which holds the claims submitted by all Czech health insurance funds. Because every procedure paid from public health insurance is captured nationally, the source provides near-complete ascertainment of screening activity irrespective of setting. Incidence data derive from the National Cancer Registry. The Registry of Preventive Colonoscopies, the National Registry of Reimbursed Health Services, and the National Cancer Registry are components of the National Health Information System, administered by the Institute of Health Information and Statistics of the Czech Republic (IHIS), whose National Screening Centre (NSC IHIS) is responsible for program monitoring. Mortality data derive from the Czech Statistical Office. Aggregate indicators and open datasets are published through the NSC data portals [13]. All analyses were performed on aggregate outputs prepared by the data custodians. No individual-level record linkage was carried out for this study, and the authors received no individual-level records.

### 2.2. Program Description

The organization of the program, and the flow of data between its registries, are shown in Figure 1. The program has evolved through the key milestones listed in Table 1, under oversight exercised by two bodies. The Committee for CRC Screening of the Ministry of Health approves program standards, and the Board for CRC Screening of the Czech Society of Gastroenterology issues clinical guidelines and accreditation criteria. Data collection, analysis, and performance reporting are the responsibility of the NSC IHIS.

Two screening pathways are offered. The first is a two-step pathway based on a biennial quantitative FIT, at an analytical cut-off of 15 µg Hb/g stool; a positive result triggers referral for FOBT-positive (FOBT+) colonoscopy. The second is screening colonoscopy, available since 2009 and repeated at 10-year intervals after a negative examination. The age threshold for screening colonoscopy was initially 55 years and was lowered to 50 in 2020. Together, FOBT+ colonoscopy and screening colonoscopy constitute preventive colonoscopy. Both colonoscopy pathways are performed exclusively in accredited Centers for Preventive Colonoscopy [13]. FIT is dispensed by general practitioners and gynecologists. Under the accreditation criteria implemented in 2025, a minimum annual volume of 100 preventive colonoscopies per center is verified from national reimbursement claims, and minimum staffing is defined in full-time equivalents. On-site audits are conducted by the 15 regional coordinators of the Board for CRC Screening [13]. Since January 2014, eligible individuals not screened within the recommended interval have received a personal invitation from their health insurance fund [14]. The target age range was 50 years and older, without an upper limit, for most of the program’s history; from 2026 it is redefined as 45–74 years.

### 2.3. Definitions and Indicators

Program coverage was measured over the standard 2-year interval in two ways. Coverage by screening modalities is the headline indicator. It measures the whole target population and is therefore related to, but not identical with, the European uptake indicator, which is calculated among invited persons. A person was counted as covered if they had completed a screening FIT during the preceding 24 months or a screening colonoscopy during the preceding 10 years; the term two-year coverage interval refers to the monitoring interval for FIT-based screening, while colonoscopy was incorporated using its recommended 10-year interval. Coverage by all methods additionally counts diagnostic colonoscopy and laboratory FIT performed outside the organized program. FIT positivity was the proportion of screening FIT results at or above the cut-off. Both pathways serve the same target population, the age threshold for screening colonoscopy having been aligned with that for fecal testing in 2020, but they differ in pre-test probability, since one follows a positive FIT. Detection was therefore reported separately for each. For screening colonoscopy, it is expressed as the adenoma detection rate (ADR) and the cancer detection rate; for FOBT+ colonoscopy, as the positive predictive value (PPV) for adenoma and for CRC. Follow-up compliance was defined as the proportion of persons with a positive FIT who underwent colonoscopy within 6 months, counting colonoscopy reported under any procedure code, not only the code specific to FOBT+ colonoscopy. Waiting time for colonoscopy is the mean interval between a positive FIT and the subsequent correctly reported FOBT+ colonoscopy. The two follow-up indicators use opposite reference points: waiting time is assigned to the period of the colonoscopy, with the preceding screening FIT identified retrospectively, and is therefore complete for 2025, whereas compliance is assigned to the period of the positive FIT and followed forward, so it is reported only for the first half of 2025. Data were extracted on 2 July 2026, with complete records to the end of 2025, so the six-month window was complete for every test included. The European standard for this step is expressed as the proportion of persons examined within 31 days rather than as an average, and that proportion was not available for the present analysis. Colonoscopy quality indicators included adequate bowel preparation and the complete colonoscopy rate defined by cecal intubation.

### 2.4. Statistical Analysis

All indicators were evaluated using basic descriptive statistics over the available observation period, depending on the specific data source. Temporal trends were summarized using annual values or, where appropriate, quarterly or half-yearly values. Regional and district-level differences were evaluated where relevant. The specific observation periods used for individual indicators are stated in the corresponding results, tables, or figures.

Incidence and mortality are expressed as age-standardized rates using the European Standard Population and are presented as annual values. Their change over time is also reported as the relative difference between three-year averages, a smoothing approach used to reduce the influence of random year-to-year variation; the periods compared are specified with each result. In addition, temporal trends in colorectal cancer incidence and mortality were assessed using Joinpoint regression analysis performed with the Joinpoint Regression Program (version 5.2.0.0, April 2024; Statistical Research and Applications Branch, National Cancer Institute, Bethesda, MD, USA). The analysis was used to identify time points at which statistically significant changes in the direction or magnitude of the temporal trend occurred. For each identified trend segment, the annual percent change (APC) was estimated to characterize the rate of change over time.

## 3. Results

### 3.1. Screening Network

In 2025 the accredited network comprised 204 centers evaluated for quality indicators, up from 199 in 2024. In 2024 the number of persons of screening age per center averaged 22,151, ranging from 13,941 to 37,138 across the 14 administrative regions. More than 4000 general practitioners held the procedure code permitting them to dispense and process quantitative FIT. Gynecologists have been approved providers since 2009. In 2025 general practitioners performed 95.5% of screening FIT and gynecologists 4.5%, and among women the shares were 92.1% and 7.9%. Women accounted for 57.2% of all persons tested.

### 3.2. Fecal Immunochemical Testing

Fecal testing evolved from guaiac chemistry through qualitative immunochemical tests to fully quantitative FIT. Both laboratory analyzers and point-of-care testing (POCT) devices are in routine use across primary care. Overall test positivity was 4.6% during the gFOBT era (2006–2010) and rose to 6–7% after the transition to FIT (2011–2016). It reached approximately 10% during the period in which quantitative POCT analyzers were introduced in 2019–2020, before declining to 8.6% nationally in 2025. The rise on transition from guaiac testing is expected: Quantitative FIT detects human hemoglobin specifically, requires no dietary restriction, and is read at a defined analytical threshold rather than by visual interpretation. Medication and other clinical factors that promote gastrointestinal bleeding may nevertheless influence positivity.

Positivity varied within the country, from 7.8% to 9.6% across administrative regions in 2025. At nominally identical cut-offs, different analyzer platforms can yield different results for the same stool sample. Mandatory annual external quality assurance for all FIT providers was introduced in 2023, with standardized control samples certified against defined tolerance limits; the first national audit following implementation indicates provider compliance of approximately 88%, leaving a minority of providers outside the scheme.

### 3.3. Colonoscopy Volumes and Detection

The Registry of Preventive Colonoscopies records each pathway separately: FOBT+ colonoscopy, performed after a positive FIT, and screening colonoscopy, performed as a first-line test without prior FIT. Between 2006 and 2025, 653,825 preventive colonoscopies were performed. At least one adenoma was detected in 251,974 of them and cancer, including carcinoma in situ, in 16,575. Findings are recorded at the level of the examination, so these counts denote examinations with a positive finding, not numbers of lesions removed. FOBT+ colonoscopies accounted for 448,862 of the total, with adenoma detected in 189,496 and cancer in 14,994, a PPV of 42.2% for adenoma and 3.3% for CRC. Screening colonoscopies, available since 2009, accounted for 204,963, with adenoma detected in 62,478 and cancer in 1581, an ADR of 30.5% and a cancer detection rate of 0.8%. The balance between the pathways has shifted: in 2025 screening colonoscopy exceeded FOBT+ colonoscopy in volume for the first time, at 32,214 against 31,051 examinations, or 50.9% of all preventive colonoscopies.

Annual volumes and proportions of adenoma and cancer findings by pathway are presented in Table 2 and Figure 2. The recorded indicators changed over time within each pathway. After a positive FIT, the PPV for adenoma rose from 29.6% to 48.3% and the PPV for CRC fell from 6.3% to 2.8%. At screening colonoscopy, the ADR rose from 25.3% in 2009 to 32.6%, and the cancer detection rate fell from 1.8% to 0.5%. Pooled across both pathways, adenoma detection rose from 29.6% in 2006 to 40.3% in 2025 and cancer detection fell from 6.3% to 1.6%; the pooled figures also reflect the changing balance between the two pathways and are therefore reported here only as a summary of overall program activity.

The total volume of colonoscopies performed for any indication grew from 173,017 procedures in 2010 to 252,309 in 2025. Screening colonoscopy and FOBT+ colonoscopy together represented 26.4% of the 2025 total (12.8% and 13.6%, respectively), compared with 8.1% in 2010, the remainder being performed for diagnostic indications. These proportions derive from reimbursement claims, which record every colonoscopy irrespective of indication, whereas the annual counts in Table 2 derive from the Registry of Preventive Colonoscopies; the two sources differ in level and should not be combined.

### 3.4. Colonoscopy Quality Indicators

Key performance indicators, with their EU reference standards and most recent national results, are presented in Table 3. Cecal intubation was achieved in 98.3% of examinations in 2025, having risen steadily from 90.8% in 2006. Adequate bowel preparation, assessed by the Boston Bowel Preparation Scale, was achieved in 97% of examinations nationally. Among examinations with an adenoma in 2025, 33.3% included an advanced adenoma, corresponding to detection of advanced adenoma in 13.4% of all preventive colonoscopies. Compliance was also assessed at center level, against six indicators: an annual volume of at least 100 preventive colonoscopies, adequate bowel preparation in at least 90% of examinations, cecal intubation in at least 95%, and adenoma detection across all preventive colonoscopies of at least 25% overall, 30% in men, and 20% in women. Of the 204 centers evaluated in 2025, five indicators were evaluable in all centers, whereas the bowel preparation indicator could be calculated for 203 centers, as it was not possible to calculate this indicator for one center. The volume threshold was met by 199 (97.5%) and cecal intubation by 193 (94.6%). The bowel preparation threshold was met by 193 of 203 centers (95.1%). The three detection thresholds were met by 188 (92.2%), 190 (93.1%), and 178 (87.3%) centers, respectively. Because one indicator could not be calculated for one center, the summary number of quality indicators met was evaluated among the 203 centers with complete information for all six indicators. All six indicators were met by 161 centers (79%), five by 21 (10%), and four by 8 (4%). The remaining 13 centers (6%) met three or fewer and are the focus of targeted quality improvement. These center-level detection thresholds are computed across both pathways together, so they are not directly comparable with the pathway-specific rates reported above. Detection was consistently higher in men, averaging 47% against 32% in women.

Adverse events are recorded in the Registry of Preventive Colonoscopies alongside the procedures and are classified by the step to which they are attributed. Among the 63,265 preventive colonoscopies performed in 2025, perforation attributed to the colonoscopy itself was reported in 51 examinations (0.08%). Polypectomy was performed in 34,253 of these examinations, with complete complication reporting in 33,856. Perforation attributed to polypectomy was recorded in 67 examinations and post-polypectomy bleeding in 564; both rates are expressed against all 34,253 polypectomies performed, giving 0.20% and 1.65%. For comparison, a meta-analysis of 82 population-based studies covering 38.5 million colonoscopies reported pooled incidences per 10,000 colonoscopies of 5.66 for perforation and 18.50 for bleeding in the European subgroup, rising to 7.37 and 52.92 during therapeutic colonoscopy, defined as colonoscopy with polypectomy [16]. A meta-analysis restricted to colorectal cancer screening reported 88 perforations and 229 severe bleeding events per 100,000 people screened for colonoscopy following a positive fecal test, and 53 and 68 per 100,000 people screened for once-only screening colonoscopy [17].

### 3.5. Program Coverage

Coverage over the standard 2-year interval is presented in Figure 3. Coverage by screening modalities reached 33.7% in 2025, the highest level recorded. It had risen from 24.4% in 2011 to 32.5% in 2015, then fell to 26.9% during the COVID-19 pandemic before recovering. FIT accounted for most of the screening coverage, with FIT-only coverage reaching 30.2% in 2025. Coverage by all methods, including also diagnostic examinations, reached 41.0% in the same year; 59.0% of the eligible population had no relevant examination within the standard interval. Between 2011 and 2025 FIT-only coverage rose from 24.2% to 30.2%, against a rise from 24.4% to 33.7% for coverage by screening modalities, so most of the increase reflects participation in fecal testing rather than the maturing ten-year look-back for screening colonoscopy. District-level coverage ranged from 24.1% to 41.6% in 2025.

The personal invitation system was launched in January 2014, initially targeting individuals aged 50–70 years who had not been examined within the standard interval. In its first year, over 1.5 million individuals (52.7% of the target population) were contacted. Coverage rose by 4.5 percentage points to a then-historical high of 30.8%. Preventive colonoscopy volume increased by 41.5% in the same year, and the numbers of adenomas and cancers detected rose by 42% and 20%, respectively, relative to 2013. Expressed per examination, adenoma detection was therefore unchanged, and cancer detection fell. The year 2014 also marks the complete phase-out of guaiac testing, so the two changes cannot be separated. Participation was 21.7% after the first invitation and declined with each successive one, to 19.2%, 14.4%, and 9.2%.

### 3.6. Follow-Up Compliance and Waiting Times

Using the predefined 6-month follow-up window, compliance with colonoscopy after a positive FIT was 58.0% for tests recorded in the first half of 2025, against 65.9% in 2010. The same estimate was obtained for the completed 2024 cohort, in which 58.0% of 61,117 persons with a positive FIT underwent colonoscopy within the window, so the half-year figure is not an artifact of incomplete observation. The examinations that are performed do not all follow the intended pathway. In the first half of 2025, the most recent period for which the breakdown was available, 58.0% of individuals with a positive FIT underwent colonoscopy, 37.6% reported under the FOBT+ code and 20.4% under a diagnostic or screening code (18.3% and 2.1%, respectively). More than one third of completed follow-up is therefore performed outside the coded FOBT+ pathway, which has implications for how the step is monitored. Completion also varied widely by district, from 42.6% to 75.5% for colonoscopy under any code, and from 6.8% to 65.7% for colonoscopy reported under the FOBT+ code.

The average waiting time between a positive FIT and FOBT+ colonoscopy was 80 days nationally in the fourth quarter of 2025. Variation across the country is substantial: in the fourth quarter of 2025, the most recent quarter with a published regional breakdown, regional means ranged from 65 to 103 days, and district means from 29 to 157 days. The national mean should therefore not be read as the waiting time experienced by a typical patient. The temporal trend shows an increase from 63 days in the first quarter of 2019 to a peak of 92 days in the third quarter of 2023, followed by a gradual decline. The rise from 2020 onwards coincided with the wider use of POCT analyzers and with a higher number of positive results; the aggregate data do not allow the contribution of either to be quantified.

### 3.7. Clinical Outcomes

Cancer was detected in 3.3% of FOBT+ colonoscopies performed between 2006 and 2025, and in 0.8% of screening colonoscopies performed between 2009 and 2025. Stage was recorded for 581 of the 1043 cancers detected in 2025. Among these, 102 (17.5%) were in situ, 213 (36.7%) stage I, 86 (14.8%) stage II, 133 (22.9%) stage III, and 47 (8.1%) stage IV, so more than half of the staged subset were in situ or stage I. Stage was not recorded for the remaining 462 cancers, 44.3% of the total, because the registry requires entry within one month of the examination, whereas definitive staging is frequently unavailable within that interval. The proportion with stage not recorded was similar or higher in earlier years: 48.7% in 2022, 48.9% in 2023 and 51.4% in 2024. The extent of missing data precludes inference about any shift in stage distribution over time. At population level, across all colorectal cancers recorded in the National Cancer Registry, the stage distribution changed only modestly over the period: stage I accounted for 16.0% of cases in 2000 and 21.3% in 2024, while cases of unknown stage fell from 16.3% to 6.8%. Successive revisions of the TNM classification during this interval limit strict comparability.

Age-standardized CRC incidence and mortality in the Czech Republic are presented in Figure 4. Both rates rose for decades and peaked around the turn of the millennium. Relative to the three-year period around the launch of the program (1998–2000), incidence in 2022–2024 was 31.7% lower and mortality 50.0% lower. Over the preceding decade (2013–2015 versus 2022–2024) the corresponding declines were 21.0% and 19.9%. Age-specific registry values, which are not plotted in Figure 4, indicate that the incidence decline was most pronounced at ages 75 and older, while the 45–49 age group, historically outside the target population, showed stagnating or mildly rising incidence. The mortality decline was concentrated in the 50–74 age group. Because the oldest age group is also the least frequently screened, this pattern is not by itself evidence of a screening effect.

Joinpoint analysis revealed distinct changes in both incidence and mortality trends (Figure 4). Age-standardized incidence increased significantly between 1977 and 1995 (APC 3.25%; 95% CI: 3.1 to 3.5) and continued to rise, although at a slower rate, between 1995 and 2002 (APC 1.24%; 95% CI: 0.4 to 2.0). The trend subsequently reversed, with a significant decline from 2002 to 2014 (APC −1.54%; 95% CI: −1.8 to −1.2), followed by a more pronounced decrease between 2014 and 2020 (APC −4.15%; 95% CI: −5.8 to −3.5). From 2020 to 2024, incidence remained relatively stable (APC 0.48%; 95% CI: −0.8 to 2.4).

Mortality increased markedly during the earlier period, although the rate of increase progressively slowed, from an APC of 24.82% in 1977–1979 to 8.57% in 1979–1982. A further increase was observed in 1982–1992 (APC 2.58%; 95% CI: −0.6 to 3.1), although this was not statistically significant. Between 1992 and 2004, mortality remained broadly stable (APC −0.16%; 95% CI: −7.4 to 0.4). A marked decline was subsequently observed in 2004–2007 (APC −7.50%; 95% CI: −8.4 to −2.4), followed by a sustained significant decrease from 2007 to 2024 (APC −2.92%; 95% CI: −3.2 to −2.6).

## 4. Discussion

The Czech program is among the longest-running national colorectal cancer screening programs in Europe, in continuous operation since 2000.

### 4.1. Principal Findings and Population-Level Trends

Across the quarter century covered by this analysis, age-standardized CRC incidence and mortality in the Czech Republic declined substantially. Joinpoint analysis showed that incidence shifted from a long-term increase to a significant decline after 2002, with the steepest decrease observed after 2014, while mortality entered a sustained downward trend from the mid-2000s onwards. These changes broadly coincided with the gradual development and strengthening of the national CRC screening program. In particular, the acceleration in the decline in incidence after 2014 temporally aligned with the introduction of mandatory FIT use and personal invitations for non-participants. However, these trends cannot be attributed to screening alone, as changes in risk factors, diagnostic practice, and treatment may also have contributed. Because individual screening histories were not linked to subsequent cancer outcomes, these data cannot establish a causal relationship; the findings are presented as concurrent program and population-level observations.

Screening was delivered at national scale: 653,825 preventive colonoscopies, with at least one adenoma detected in 251,974 examinations and cancer, including carcinoma in situ, in 16,575. Recorded detection indicators rose over the period: the PPV for adenoma after a positive FIT from 29.6% to 48.3%, and the ADR at screening colonoscopy from 25.3% in 2009 to 32.6%. Cancer was found in a decreasing proportion of examinations in both pathways, from 6.3% to 2.8% and from 1.8% to 0.5%, a pattern that may reflect repeated screening, changes in testing practice, or changes in the composition of the screened population; their relative contributions cannot be separated. In 2025 screening colonoscopy accounted for 50.9% of preventive colonoscopies and exceeded FOBT+ colonoscopy in annual volume for the first time, a change in pathway composition that should be considered when pooled detection indicators are interpreted over time. At the population level, incidence in 2022–2024 was 31.7% lower and mortality 50.0% lower than in the three-year period around the launch of the program, a comparison that spans a longer interval than the colonoscopy registry series. Age-specific patterns were heterogeneous: the incidence decline was most pronounced at ages 75 and older, the mortality decline was concentrated at ages 50–74, and incidence at ages 45–49 was stagnating or mildly rising. Because the oldest age group is also the least frequently screened, these patterns are not by themselves evidence of a screening effect.

The timing of the two trends nevertheless deserves closer attention than a summary decline conveys. Half of the mortality reduction recorded between 2000 and 2024, derived from the annual series plotted in Figure 4, had already occurred by 2010, when the registry documents 54,268 preventive colonoscopies, 8.3% of the 2006–2025 total. Incidence behaved differently: only 16% of its decline preceded 2010, the remainder occurring in the years of substantially larger recorded volumes of preventive colonoscopy. This divergence argues against attributing both trends to screening. Concurrent changes in surgical technique, systemic therapy, and the organization of oncological care over the same quarter century may also have contributed, and their relative contributions cannot be separated in the present data. The magnitude of any contribution of screening to either trend cannot be estimated from aggregate data. At the population level, only a modest increase in stage I disease was recorded, from 16.0% of cases in 2000 to 21.3% in 2024, alongside a fall in the proportion with unknown stage; successive revisions of the TNM classification and changing completeness of stage recording limit comparison, and no stage shift can be attributed to screening.

International experience shows both a similar direction and a similar temporal pattern. Across 21 European countries, substantial sustained declines in incidence were observed in Austria, the Czech Republic, and Germany, all of which operate long-standing programs [18]. In a meta-analysis of 58 organized programs, the largest pooled mortality reduction, 41.8%, was recorded in the heterogeneous group of programs offering a choice between fecal occult blood testing and colonoscopy, and programs operating for more than five years were associated with lower CRC mortality [19]. Germany introduced screening colonoscopy in 2002 and shows a parallel pattern of falling incidence and mortality, at a cumulative uptake of only 20–25% [20]. After implementation of the Dutch FIT-based program, an initial rise in incidence was followed by declines in overall and advanced-stage incidence and in mortality [21]. This sequence is a useful European comparator, but it is not a signature by which the Czech trends could be attributed to screening.

### 4.2. Coverage in the European Context

Coverage remains a major implementation gap, alongside completion of colonoscopy after a positive FIT. The Czech coverage measure and the European uptake benchmark rest on different denominators and are therefore not directly equivalent: the European figure is an uptake rate among invited persons, whereas the Czech measure is coverage of the whole target population within the standard interval. Numerically, the 2025 coverage of 33.7% is below the 45% acceptable uptake benchmark cited in the European guidelines [3]. A comparison of 18 European programs documented a median examination coverage of only 24.1% [22], so the Czech value lies within a wide and heterogeneous European range. Differences in target age, screening interval, and indicator definition nevertheless limit direct ranking. The 2026 European Court of Auditors report identified continuing weaknesses in the implementation, sustainability, and monitoring of cancer screening across the European Union [10]. The broader all-methods measure of 41.0% counts examinations performed for clinical indications and is therefore not comparable with a screening uptake standard either. It should therefore be read as a measure of population exposure to relevant examinations rather than as screening participation, a distinction documented across Europe by health-interview data [11]. Czech analyses have quantified coverage by all examinations, including those performed outside the organized program, and its association with the detection of early colorectal neoplasia [14]. The target population comprised approximately 4.5 million persons in 2025. The numerical distance between 33.7% and 45% should therefore not be read as a directly quantifiable implementation gap; both indicators nonetheless point to substantial scope for increasing participation. Regional piloting of direct home mailing of test kits is the measure adopted to that end.

Substantial differences relative to the leading programs persist. The figures that follow are not uniformly defined: rates reported among invited persons are not directly comparable with the Czech population-coverage measure, whereas figures expressed as a share of the target population are closer in concept. They indicate the range of European program performance rather than a ranking. Across European programs the reported values span a wide range, from approximately 8% to about 79% [23,24,25,26,27,28,29,30]. Values reported among invited or referred persons lie at the upper end. The Netherlands, for example, reports 67.1% participation, with 84.7% colonoscopy uptake among those referred [23]. Values expressed as a share of the target population are closer in concept to the Czech measure. France reports 30.7% on that basis [27], and a European synthesis reports approximately 65% in Slovenia and 8% in Hungary [28], both programs being described in more detail elsewhere [29,30]. Denmark, England and Finland report values between these two conventions [24,25,26]. The observed differences may reflect variation in program design, invitation strategy, target populations, indicator definitions, political context, and other unmeasured factors. One plausible structural contributor is well documented. Test distribution historically required an in-person encounter with a general practitioner, which may represent a structural barrier to participation, particularly for individuals with limited contact with health services. Across Europe, direct home mailing of test kits has been identified as one implementation feature that may facilitate access to organized screening [11]. Czech participation after repeated personal invitations declined from 21.7% after the first invitation to 9.2% after the fourth, which identifies persistently unscreened individuals as the group for which alternative approaches are required, although no comparison between invitation strategies can be made from these data.

### 4.3. FIT Performance, Follow-Up Compliance, and Waiting Times

The FIT positivity threshold is a major determinant of positivity, positive predictive value, colonoscopy demand, and yield, each of which is also influenced by population characteristics, screening round, and analytical platform. The Czech cut-off of 15 µg Hb/g places the program at the sensitive end of European practice. That practice extends from 20 µg/g in Denmark [24] through 30 µg/g in France [31] to 120 µg/g in England [32]. Czech positivity of 8.6% is higher than in the Netherlands [23] and above the 5.3–6.6% range summarized across mature European FIT programs [33], although cross-program differences should not be attributed to the threshold alone. Regional positivity ranged from 7.8% to 9.6% in 2025. It should likewise not be read as a difference in population risk alone: several analyzer platforms operate at a nominally common cut-off, and participation in the first national external quality assessment was approximately 88%. The prevailing trend is nevertheless toward greater sensitivity. Following a UK National Screening Committee recommendation, England began in January 2026 to lower its threshold from 120 to 80 µg Hb/g, with the phased rollout due for completion by March 2028 [34]; modeling suggests that further reduction would yield additional benefit at the cost of substantially greater colonoscopy demand [32]. The Dutch experience is instructive for capacity-constrained programs. When referrals exceeded endoscopic capacity, the cut-off was raised from 15 to 47 µg Hb/g; positivity fell, predictive value improved, and much of the neoplasia initially missed was detected in the subsequent round [35]. The operational consequences of the Czech threshold depend on local positivity, follow-up completion, and available colonoscopy capacity, and the present aggregate analysis cannot identify an optimal threshold for the Czech program. Detection indicators restricted to 2023 onwards, when quantitative FIT at a standardized cut-off was mandatory and external quality assurance was in place, give an adenoma detection rate at screening colonoscopy of 31.9% and a positive predictive value for adenoma after a positive FIT of 47.1%. Both are close to the 2025 values, so the rise recorded across the full period reflects the change in test technology and in reporting as much as a change in endoscopic performance. Whether providers outside the external quality assurance scheme differ systematically in test volume or reported positivity could not be determined from the available aggregate data.

Completion of colonoscopy after a positive test represents the largest documented deficit in the screening pathway. Czech compliance of 58.0% is among the lowest reported by European programs. An identical estimate was obtained for the completed 2024 cohort, so the figure does not depend on the length of observation available for the most recent tests. Cross-country comparisons should also be interpreted cautiously because of heterogeneity in definitions, target populations, and reporting intervals. The Czech figure counts colonoscopy under any code, and more than one third of completed follow-up is reported outside the dedicated FOBT+ code, so the organized pathway captures less of the activity than the headline figure implies, and pathway-specific detection and quality indicators may omit or misclassify some colonoscopies performed after a positive FIT. Completion also ranges from 42.6% to 75.5% between districts, marked geographic heterogeneity whose determinants cannot be identified from the aggregate data. The cross-country median for further-assessment participation is 81.1% [22]; England reports 76.0% uptake of diagnostic testing, defined as colonoscopy or computed tomographic colonography [25], and the Netherlands 84.7% [23], against the European guideline standard of at least 85% [3]. The contrast with Denmark is particularly instructive. Compliance there approximates 90%, and colonoscopy is scheduled within 14 days of a positive FIT [24]. The Czech national mean was 80 days in the fourth quarter of 2025. In the fourth quarter of 2025, the most recent period with a geographic breakdown, regional means ranged from 65 to 103 days and district means from 29 to 157 days, a spread comparable to that observed for follow-up completion. The European standard is expressed as the proportion of persons examined within 31 days of referral rather than as an average, so the two quantities are not directly comparable, and the proportion meeting the 31-day standard was not available here [3]. The waiting-time result should therefore be read as a descriptive indicator of pathway duration rather than as an estimate of compliance with the European time standard. Waiting time and follow-up completion are reported here as parallel indicators of pathway performance; because screening histories were not linked at the individual level, no relationship between them can be established from these data. Compliance and the interval to colonoscopy are recognized as the foremost operational priorities and are addressed as separate implementation targets.

### 4.4. Colonoscopy Quality, Safety, and the Randomized Evidence

The adenoma detection rate is the endoscopist-level indicator most robustly associated with clinical outcomes. In the Polish program, patients of endoscopists with an ADR below 11% had a hazard ratio for interval CRC of 10.94 (95% confidence interval 1.37–87.01), relative to those examined by endoscopists with an ADR of 20% or higher [36]. An endoscopist’s improvement in ADR is associated with lower interval-cancer incidence and mortality [37]. After a single negative screening colonoscopy, CRC incidence and mortality were 72% and 81% lower than in the general population over up to 17.4 years [38]. Only high-quality examinations sustained that protection beyond ten years. This evidence underpins the European Society of Gastrointestinal Endoscopy (ESGE) performance measures (adequate bowel preparation ≥ 90%, cecal intubation ≥ 90%, and ADR ≥ 25%), which the Czech accreditation criteria incorporate, applying a higher cecal intubation threshold of 95% [12].

Czech performance against these benchmarks has improved over time. The 2025 ADR at screening colonoscopy, 32.6%, exceeds the ≥25% minimum standard of the European performance measures [12]. It remains below the ≥35% threshold adopted by the 2024 quality indicators of the American Society for Gastrointestinal Endoscopy and the American College of Gastroenterology, which was revised upward as post-colonoscopy CRC risk continues to decline with rising detection rates [39]. The FOBT+ PPV for adenoma, 42.2% cumulatively and 48.3% in 2025, exceeds the 34.4% pooled real-world estimate for colonoscopy after a positive fecal test [15], which is a meta-analytic average rather than a normative standard. Performance also varied between centers. Among the 203 centers for which all six indicators could be calculated in 2025, 161 (79%) met all six accreditation indicators, whereas 13 (6%) met three or fewer, so national averages conceal a small group of centers for which targeted quality improvement is the more immediate priority. Detection differed by sex, averaging 47% in men against 32% in women, a difference the accreditation criteria accommodate through separate thresholds of 30% and 20%, and which is considered further among the limitations below. Advanced adenoma was detected in 13.4% of preventive colonoscopies in 2025. Among the 581 cancers with stage recorded that year, 315 (54.2%) were in situ or stage I; because stage was missing for 44.3% of the cancers detected, this distribution is descriptive only and cannot be used to infer a stage shift over time. Perforation attributed to the colonoscopy itself was 8.1 per 10,000 examinations; this is not directly comparable with the screening meta-analysis, which reported risk per 100,000 people screened rather than per completed colonoscopy [17]. The rates attributed to polypectomy, 19.6 perforations and 165 bleeding events per 10,000 polypectomies performed, are higher than the pooled values for therapeutic colonoscopy [16]. Ascertainment differs: the Czech figures come from mandatory procedure-level reporting for a single year, without stratification by procedural complexity, whereas the pooled estimates rest largely on administrative and registry sources across heterogeneous settings. The 2025 values should therefore be read as descriptive surveillance data rather than as comparative estimates of procedural safety. Post-colonoscopy CRC rates could not be estimated, because no individual-level linkage between colonoscopy and cancer registration was available for this analysis. The Dutch program reported a 3-year post-colonoscopy CRC rate of 2.7%, calculated according to the World Endoscopy Organization definition, after colonoscopy following a positive FIT [40]. A national audit conducted according to the World Endoscopy Organization methodology is the logical next step beyond ADR monitoring and is planned as a separate analysis.

Randomized evidence frames these observations. The Nordic-European Initiative on Colorectal Cancer (NordICC) is the only pragmatic trial of screening colonoscopy versus no screening. At 13 years it showed a 19% reduction in CRC incidence in the intention-to-screen analysis and 45% per protocol, whereas CRC mortality did not differ significantly, despite point estimates corresponding to reductions of 12% and 30%, respectively [41]. In the Spanish COLONPREV trial, invitation to biennial FIT was non-inferior to invitation to one-time colonoscopy for CRC mortality at ten years, a result the investigators relate to substantially higher participation in the FIT arm [42]. Together these trials indicate that program-level outcomes depend on both test performance and participation. Several programs that mail FIT kits directly have reported uptake above 60% [43], against participation in any screening of 39.9% in the FIT arm and 31.8% in the colonoscopy arm of COLONPREV [42]. For the Czech program, the available indicators suggest that participation and follow-up after a positive FIT may represent more important implementation constraints than measured endoscopic performance, which exceeds the applicable European minimum benchmarks.

### 4.5. Program Developments and Outlook

A major program change implemented in 2026 is the redefinition of the target age range as 45–74 years [13]. Before this change the Czech Republic was one of the few EU member states without an explicit upper age limit. The new lower bound is consistent with several international recommendations and national programs, although recommended starting ages remain heterogeneous across jurisdictions. The US Preventive Services Task Force lowered the recommended starting age to 45 in 2021 [44], and the Austrian National Committee for Cancer Screening has recommended a nationwide program for adults aged 45–75 [45]. The 2022 EU Council Recommendation nevertheless retains 50–74 as the reference range [46]. Incidence of early-onset CRC is rising across Europe and North America [47]. In Taiwan, biennial FIT begun at ages 40–49 was associated with 21% lower CRC incidence and 39% lower mortality than starting at 50 [48], and US registry data showed a marked increase in localized CRC diagnoses at ages 45–49 after recommendations for earlier screening, a pattern that may reflect earlier detection [49]. Czech data likewise document stagnating incidence at ages 45–49. The new upper bound of 74 also aligns the Czech target population with the bounded age ranges used in European recommendations and programs.

Further developments address the organizational constraints identified above. A national monitoring platform is under development to replace batch processing. It is intended to support more timely invitation tracking, practice-level coverage monitoring, linkage of the FIT and colonoscopy steps, and reporting of median and percentile-based waiting times, which are directly interpretable against the European 31-day standard. More centralized FIT processing is one structural option for reducing the inter-platform analytical variability documented here. The 2025 European Society of Gastrointestinal Endoscopy position statement summarized randomized evidence in which computer-aided detection increased the ADR by 8 percentage points (95% confidence interval 6 to 11) and issued a weak recommendation in favor of its use, noting low certainty of evidence and the additional burden of increased polyp detection and surveillance [50]. Evidence on computer-aided diagnosis is less favorable. Two meta-analyses of real-time optical diagnosis of diminutive polyps found no incremental benefit and no incremental harm from computer-aided diagnosis relative to unassisted endoscopist prediction, which leaves its value in the resect-and-discard strategy unresolved [51,52]. It is being piloted in leading Czech centers. A new generation of stool- and blood-based tests may further broaden the reach of organized programs [53].

### 4.6. Strengths and Limitations

The principal strength of this study is the use of long-running, nationally comprehensive registry and reimbursement data covering the 2006 to 2025 period of a program in continuous operation since 2000. Although the underlying national information infrastructure permits record linkage for selected monitoring purposes, the present study was based exclusively on aggregate data and did not link individual screening histories to cancer incidence, stage, or mortality outcomes. Its limitations are those inherent to comparisons of aggregate, population-level data across countries. Such data come from different periods and use different definitions; English 30-month coverage among ages 60–74 [25], for example, is not the Czech standard-interval measure. Cross-country differences of a few percentage points should therefore be read as indicative. One difference is systematic rather than incidental: Czech coverage is measured among all individuals aged 50 and over, without an upper limit, whereas the European standard and most comparator programs apply to bounded ranges, typically 50 to 74 years. Including older individuals, who are screened less often, lowers the Czech figure relative to programs reporting on a bounded population. Together with the different denominator, this means that the 33.7% coverage should not be read as a directly quantifiable shortfall from the 45% uptake benchmark. The coverage indicator also incorporates screening colonoscopy over a ten-year look-back. Because screening colonoscopy was introduced in 2009, that component reached its full observation window only in 2019, so part of the earlier rise in coverage reflects maturation of the indicator rather than increased participation. Program-level colonoscopy indicators are available from 2006 and coverage estimates from 2011, so the period from 2000 to 2005 is not represented by registry-based performance indicators and the early phase of the program is less completely characterized than the later period.

Several clinical and methodological constraints apply. Post-colonoscopy colorectal cancer is not monitored systematically in the program, so the outcome for which the adenoma detection rate serves as a surrogate cannot itself be reported; establishing such monitoring is the most important next step in program evaluation. Linkage of the Registry of Preventive Colonoscopies to the National Cancer Registry would in addition recover the stage information missing for cancers detected at colonoscopy, and is the natural complement to that monitoring. Waiting time is reported as a national mean, which does not characterize the distribution or its upper tail; median and percentile-based measures were not available for the present analysis. A further limitation is that a single FIT threshold is applied to both sexes, although hemoglobin concentrations in stool differ systematically between men and women; a uniform cut-off may therefore result in different sensitivity, specificity, and referral burden between men and women, and the 2022 EU Council Recommendation explicitly allows risk-adapted thresholds [46]. Sex differences in positivity, detection, and referral burden should accordingly not be read as reflecting disease prevalence or endoscopic performance alone. Complication rates are reported for a single year and are not stratified by procedural complexity, polypectomy technique, or lesion characteristics, so they are descriptive surveillance data rather than comparative safety estimates. Center-level compliance with the detection thresholds could not be reported separately by screening pathway, because the national monitoring outputs are not currently produced in that form. Stage was missing for 44.3% of the cancers detected in 2025, which precludes any analysis of stage shift, the mechanism through which screening would be expected to act on mortality.

Finally, program effectiveness was not estimated in this study; the Czech observations are population-level trends rather than a randomized comparison. Screening histories were not linked to subsequent cancer outcomes, and no counterfactual or control population was used. The contribution of the program therefore cannot be separated from concurrent changes in treatment and case management, in diagnostic practice and cancer coding, in the prevalence of risk factors, in testing performed outside the organized program, or from the disruption caused by the COVID-19 pandemic. The joinpoints identify changes in the slope of the population-level trends but not their causes, so they should not be read as evidence of a screening effect. Mortality also fell proportionally more than incidence, a pattern compatible with concurrent improvements in outcomes after diagnosis, which further limits interpretation of the mortality trend in relation to screening.

## 5. Conclusions

Twenty-five years after its launch, the Czech National CRC Screening Program has delivered screening at national scale. The period of its operation coincided with a substantial improvement in the national CRC burden. The two trends differed by age, the incidence decline being most pronounced at ages 75 and older and the mortality decline concentrated at ages 50 to 74, so their coincidence with sustained screening is not by itself evidence of a screening effect. Age-standardized incidence is 31.7% lower and mortality 50.0% lower than in the three-year period around the launch of the program, and Czech incidence has converged toward the European average [2]. The program accumulated 653,825 preventive colonoscopies over 2006–2025, with at least one adenoma detected in 251,974 examinations and cancer, including carcinoma in situ, in 16,575.

The priorities for the next phase lie in the organization of the screening pathway rather than in endoscopic performance. Coverage of the whole target population within the standard interval was 33.7%; although this is not directly equivalent to the European uptake benchmark among invited persons, both indicators point to substantial scope for improving participation. Six-month compliance with follow-up colonoscopy was 58.0%, counted under any procedure code; the remaining 42.0% of people with a positive FIT had no colonoscopy within that window. Inter-platform FIT variability limits comparability. Each is monitored nationally and each is being addressed: coverage through regional piloting of direct home mailing, follow-up through measures to shorten the interval to colonoscopy, and analytical variability through more centralized FIT processing. The 2026 revision of the target age range serves a different purpose, and because it redefines the age range on which the coverage indicator is calculated, coverage figures from 2026 will not be directly comparable with the series reported here. These are the dimensions in which ground remains to be gained. They are the priority indicators for prospective evaluation of program performance.

## Figures and Tables

**Figure 1:**
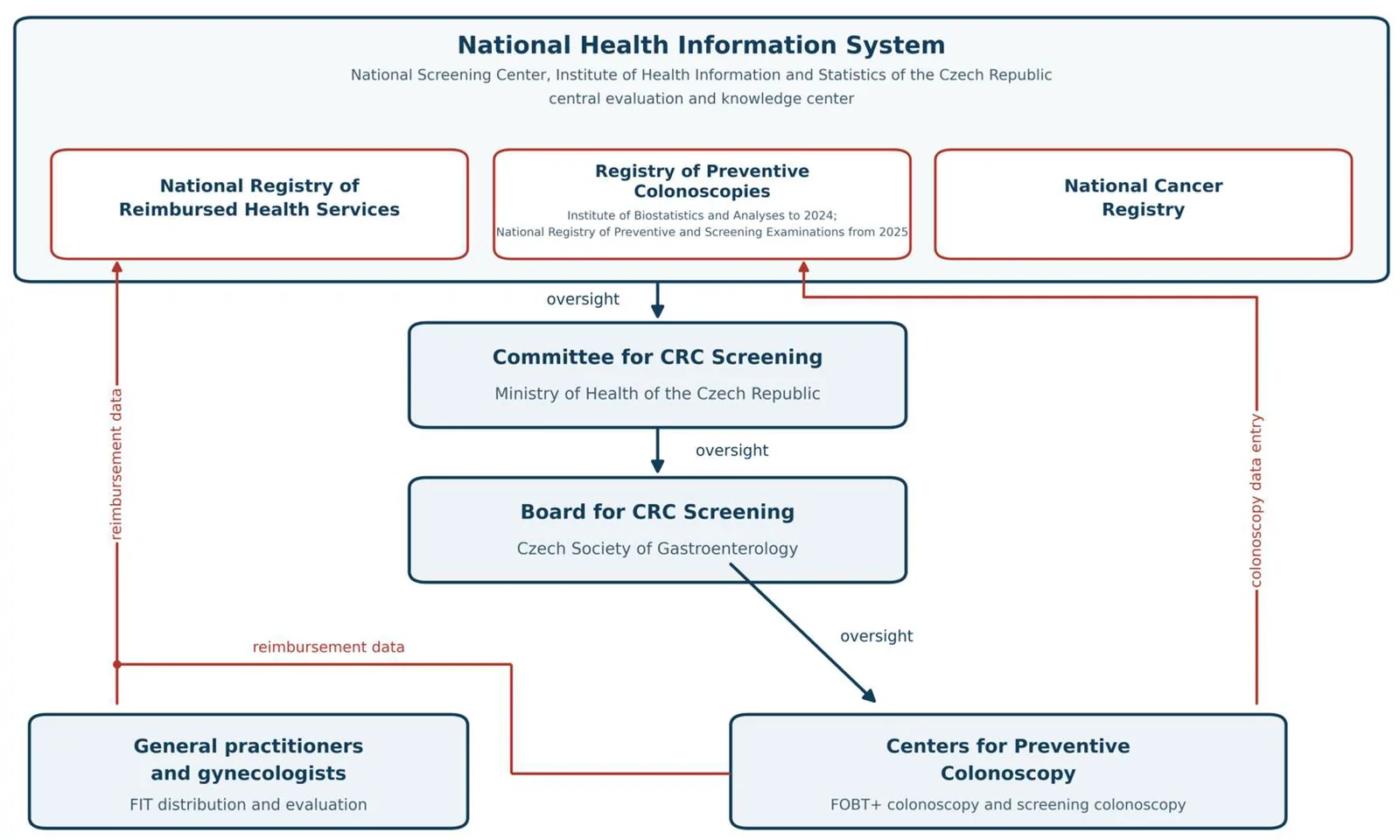
Organization and data flow of the Czech National Colorectal Cancer Screening Program. CRC = colorectal cancer; FIT = fecal immunochemical test; FOBT+ = colonoscopy after a positive fecal occult blood test; NSC IHIS = National Screening Centre of the Institute of Health Information and Statistics of the Czech Republic. Source: authors’ scheme based on NSC IHIS program documentation.

**Figure 2:**
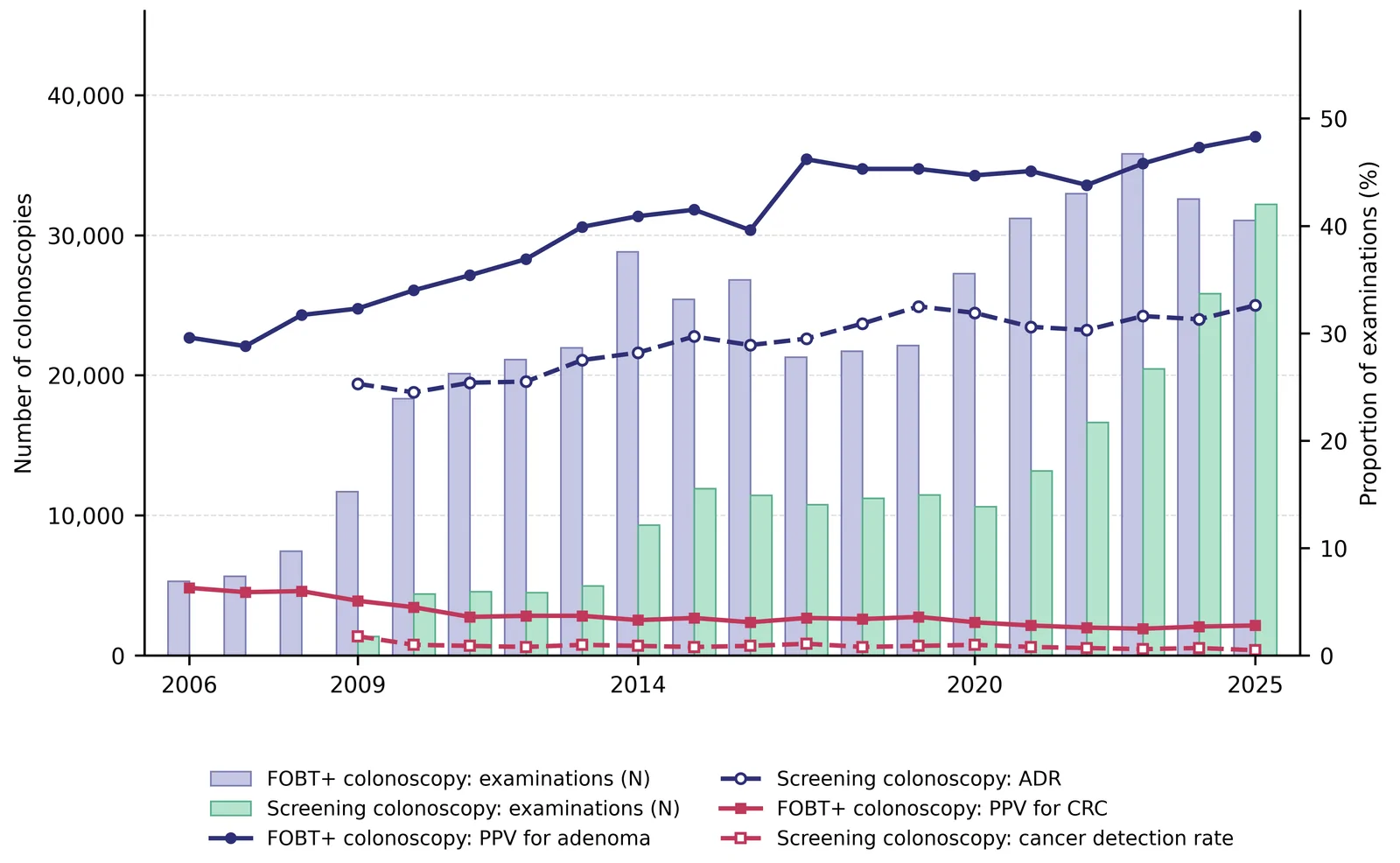
Preventive colonoscopy volumes and detection indicators by pathway, 2006–2025. Labeled years mark the introduction of screening colonoscopy (2009), the launch of the personal invitation system (2014), and the lowering of the age threshold for screening colonoscopy to 50 years (2020). ADR = adenoma detection rate; CRC = colorectal cancer; FOBT+ = colonoscopy after a positive fecal occult blood test; PPV = positive predictive value; NSC IHIS = National Screening Centre of the Institute of Health Information and Statistics of the Czech Republic. Data source: NSC IHIS and Institute of Biostatistics and Analyses, unpublished registry export of 26 February 2026.

**Figure 3:**
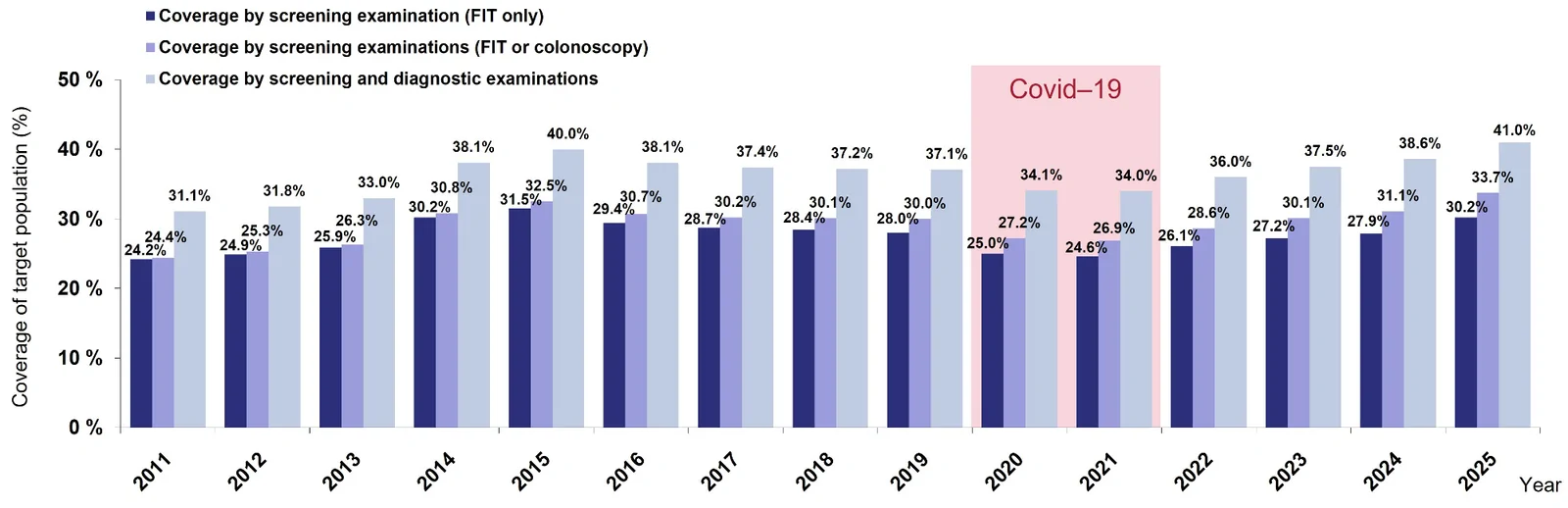
CRC screening program coverage within the applicable screening intervals among individuals aged 50 years and older, 2011–2025. FIT was assessed over a 24-month look-back and screening colonoscopy over a 10-year look-back. CRC = colorectal cancer; EU = European Union; NSC IHIS = National Screening Centre of the Institute of Health Information and Statistics of the Czech Republic. Data source: National Registry of Reimbursed Health Services, NSC IHIS.

**Figure 4:**
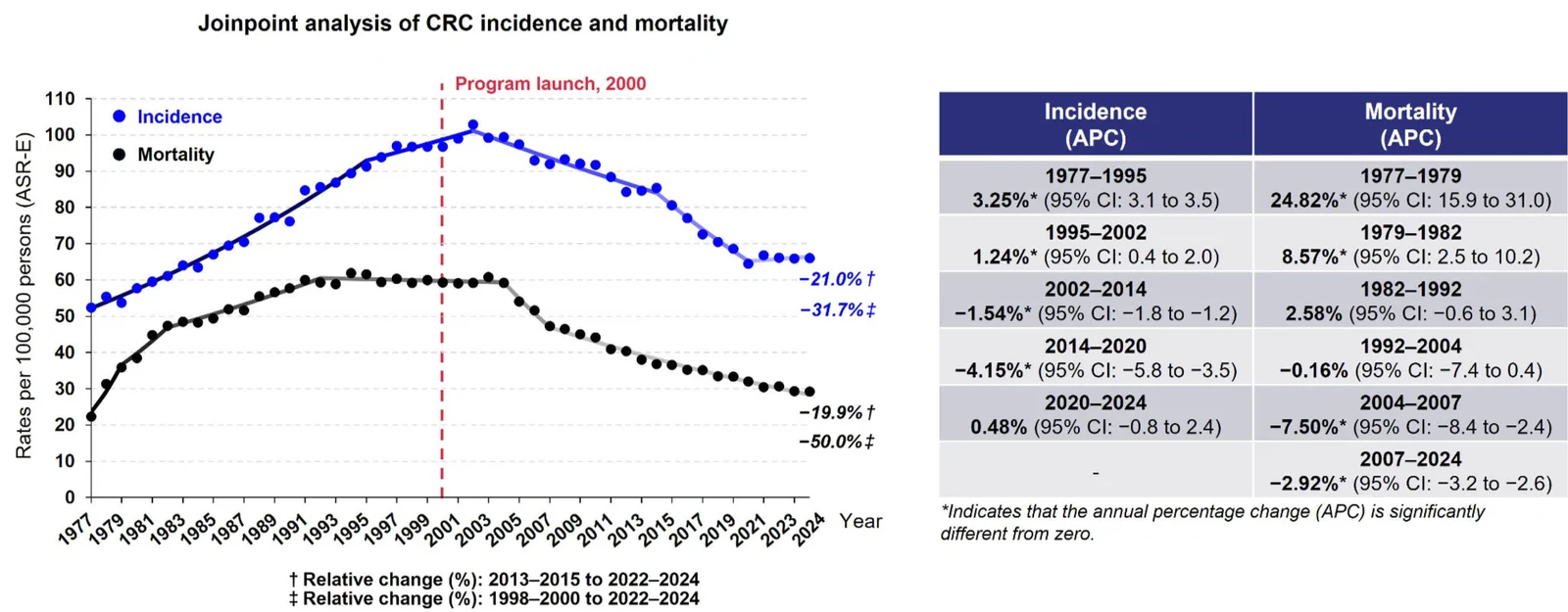
Joinpoint analysis of age-standardized CRC incidence and mortality, Czech Republic, 1977–2024. Points represent observed annual age-standardized incidence and mortality rates, and solid lines represent the fitted joinpoint regression models, with changes in slope corresponding to model-identified joinpoints. Annual percentage changes (APCs) and their 95% confidence intervals for individual trend segments are shown in the accompanying table; an asterisk indicates an APC significantly different from zero. The red dashed vertical line marks a point in time and does not represent a modeled counterfactual. Percentage labels indicate relative changes between the three-year averages for 2013–2015 and 2022–2024, and between 1998–2000 and 2022–2024, marked † and ‡ respectively. ASR-E = age-standardized rate, European standard population, per 100,000 inhabitants; CRC = colorectal cancer; APC = annual percentage change. IHIS = Institute of Health Information and Statistics of the Czech Republic. Data source: National Cancer Registry, IHIS (incidence) and Czech Statistical Office (mortality).

**Table 1:** Relevant key milestones in the program development, 2000–2026.

Year	Milestone
2000	National CRC Screening Program launched. gFOBT (Haemoccult) biennially by GPs for age ≥ 50. Positive result → referral for colonoscopy [9].
2001	Board for CRC Screening of the Czech Society of Gastroenterology established. Regional coordinators selected. Endoscopic centers mapped [13].
2003	Committee for CRC Screening formally appointed by the Ministry of Health [13].
2007	Registry of Preventive Colonoscopies launched (2006 records entered retrospectively). Transition from paper-based to individual online data collection. kolorektum.cz public portal opened [9,13].
2009	Both gFOBT and qualitative FIT permitted. Gynecologists added as approved FIT providers. Screening colonoscopy introduced for age ≥ 55 [9,13].
2010	Qualitative FIT becomes predominantly used in practice [13].
2014	FIT made mandatory and gFOBT (Haemoccult) phased out completely, with quantitative measurement recommended. Personal invitation system launched in January for individuals aged 50–70 not screened within the recommended interval [9,13].
2020	Screening colonoscopy age threshold lowered from 55 to 50 years. Quantitative FIT only, on point-of-care or laboratory analyzers at a cut-off of 15 µg Hb/g stool [13].
2023	Mandatory annual external quality assurance (EQA) for FIT. Standardization of FIT positivity cut-off [13].
2024	Updated colonoscopy standards and accreditation criteria [13].
2026	Target age range redefined as 45–74 years for both FIT and screening colonoscopy [13].

Data sources: Czech program documentation [9,13]. Abbreviations: CRC = colorectal cancer; EQA = external quality assurance; FIT = fecal immunochemical test; gFOBT = guaiac fecal occult blood test; GP = general practitioner.

**Table 2:** Annual preventive colonoscopy volumes and proportions of adenoma and cancer findings by pathway, 2006–2025.

Year	FOBT+ Colonoscopy			Screening Colonoscopy		
	N	Adenoma (%)	Cancer (%)	N	Adenoma (%)	Cancer (%)
2006	5319	29.6	6.3	—	—	—
2007	5677	28.8	5.9	—	—	—
2008	7459	31.7	6.0	—	—	—
2009	11,710	32.3	5.1	1362	25.3	1.8
2010	18,330	34.0	4.5	4411	24.5	1.0
2011	20,132	35.4	3.6	4577	25.4	0.9
2012	21,138	36.9	3.7	4496	25.5	0.8
2013	21,973	39.9	3.7	4970	27.5	1.0
2014	28,820	40.9	3.3	9313	28.2	0.9
2015	25,421	41.5	3.5	11,923	29.7	0.8
2016	26,806	39.6	3.1	11,447	28.9	0.9
2017	21,297	46.2	3.5	10,768	29.5	1.1
2018	21,724	45.3	3.4	11,221	30.9	0.8
2019	22,135	45.3	3.6	11,475	32.5	0.9
2020	27,271	44.7	3.1	10,637	31.9	1.0
2021	31,217	45.1	2.8	13,196	30.6	0.8
2022	32,971	43.8	2.6	16,645	30.3	0.7
2023	35,817	45.8	2.5	20,469	31.6	0.6
2024	32,594	47.3	2.7	25,839	31.3	0.7
2025	31,051	48.3	2.8	32,214	32.6	0.5
Total	448,862	42.2	3.3	204,963	30.5	0.8

Adenoma (%) and Cancer (%) denote the proportion of examinations in which at least one adenoma, or a cancer, was detected; the corresponding counts are examinations with a positive finding, not numbers of lesions. Cancer includes carcinoma in situ. Data source: Registry of Preventive Colonoscopies, export of 26 February 2026 (2006–2024, Institute of Biostatistics and Analyses; from 2025, National Registry of Preventive and Screening Examinations, NSC IHIS). Screening colonoscopy has been available since 2009. Screening coverage and screening-FIT volumes fell during the 2020–2021 COVID-19 pandemic; preventive colonoscopy volumes continued to rise. Abbreviations: FIT = fecal immunochemical test; FOBT+ = colonoscopy after a positive fecal occult blood test; NSC = National Screening Centre; IHIS = Institute of Health Information and Statistics of the Czech Republic.

**Table 3:** Key performance indicators (KPIs) for the program: external reference standards and benchmarks, Czech target standards, and most recent national results.

Indicator	External Reference or Benchmark	Czech Acceptable Standard	Period	National Result
Population coverage by organized screening modalities (standard interval)	>45% uptake among invited persons (acceptable standard) [3] †	≥30%	2025	33.7%
Program coverage—all methods (standard interval)	—	—	2025	41.0%
FIT positivity rate	—	—	2025	8.6% (range 7.8–9.6% by region)
Compliance with follow-up colonoscopy after positive FIT §	≥85% (desirable ≥90%) [3]	≥80%	First half of 2025	58.0%
Complete colonoscopy rate (cecal intubation)	>90% (desirable >95%) [3]	≥95%	2025	98.3%
Adequate bowel preparation (Boston Bowel Preparation Scale)	≥90% [12]	≥90%	2025	97%
Adenoma detection rate (screening colonoscopy)	≥25% [12]	≥25% (center level; ≥30% men, ≥20% women)	2025	32.6%
Positive predictive value for adenoma (FOBT+)	34.4% pooled real-world estimate [15]	—	2025	48.3%
Cancer detection rate (screening colonoscopy)	—	Monitored	2025	0.5%
Positive predictive value for cancer (FOBT+)	—	Monitored	2025	2.8%
Waiting time for FOBT+ colonoscopy after positive FIT (national mean) ‡	≤31 days in ≥90% of cases [3] ‡	≤90 days	Fourth quarter of 2025	80 days

† The European figure is an uptake rate among invited persons; the Czech figure is coverage of the whole target population within the standard interval, so the two are related but not identical. § Compliance is measured over a 6-month follow-up window, which was complete for every test included; the same estimate was obtained for the completed 2024 cohort (Section 3.6). ‡ The European standard is expressed as the proportion of persons examined within 31 days; the Czech figure is a national mean, and the proportion meeting the 31-day standard was not available for the present analysis, so the two are not directly comparable. Data sources: current-period indicators, NSC IHIS; detection indicators, Registry of Preventive Colonoscopies and National Registry of Preventive and Screening Examinations. Abbreviations: ADR = adenoma detection rate; FIT = fecal immunochemical test; FOBT+ = colonoscopy after a positive fecal occult blood test; NSC = National Screening Centre; IHIS = Institute of Health Information and Statistics of the Czech Republic.

## Data Availability

Program coverage, screening test volumes, follow-up compliance, waiting times, and national incidence and mortality are openly available from the National Screening Centre of the Institute of Health Information and Statistics of the Czech Republic (IHIS) at https://nsc-data.uzis.cz and https://www.kolorektum.cz/, and are updated annually. The annual colonoscopy volumes and detection indicators by pathway presented in Table 2 and Figure 2 derive from an aggregate export of the Registry of Preventive Colonoscopies dated 26 February 2026, which is not part of the published open datasets; these aggregate data are available from the corresponding author upon reasonable request, subject to the permission of IHIS and to applicable data-governance requirements. No new primary data were created in this study.
